# Lightweight 3D‐net Copper‐Plated Polyimide Current Collector for Lithium‐Ion Batteries

**DOI:** 10.1002/open.202400018

**Published:** 2024-11-26

**Authors:** Tingyu Song, Yonggang Min

**Affiliations:** ^1^ School of Materials and Energy Guangdong University of Technology Guangzhou 510006 China

**Keywords:** 3D-net current collector, Polyimide, Electroless plating, copper, Lithium-ion battery

## Abstract

Current collector (CC) is an indispensable constituent in lithium‐ion battery (LIB). It plays an important role in supporting electrode materials and conducting electrons between the electrode materials and the external circuit. However, the density of metal CC is high and it hinders the lightweight development of LIB. In this work, lightweight three‐dimensional (3D) CC was designed and prepared via a facile electroless plating method. The 3D copper‐plated polyimide CC has a low areal density. The 3D structure of Cu‐PI CC 2 can facilitate Li^+^ transfer between electrode materials and electrolyte owing to electrolyte can permeate through electrode. Moreover, the lithium titanate anode assembled with the 3D copper‐plated polyimide Cu‐PI CC 2 exhibited enhanced cycling and rate performance.

## Graphical abstract

In this work, 3D‐net copper‐plated polyimide current collector was prepared through electroless plating method and obtained a structure of PI core and Cu shell. This novel current collector possesses a lightweight areal density. Three different Cu loading copper‐coated PI CC samples was prepared and compared. The lithium‐ion batteries assembled with Cu‐PI CC 2 exhibit better performance.

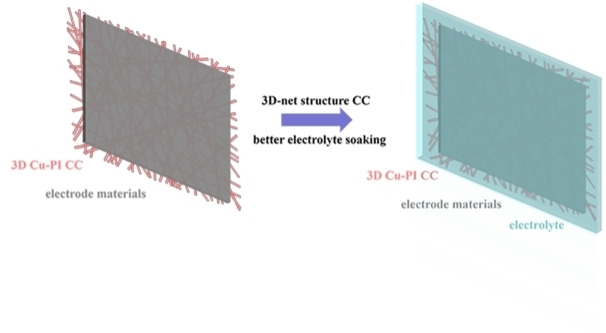

## Introduction

LIB has been widely applying in electronic vehicles and portable electric devices. CC, as an indispensable constituent of LIB, plays an important role in electron conduction and mechanical support for electrode materials.^[1]^ Copper foil has been used as CC for anode and Al foil for cathode. Cu and Al foil CCs have the double advantages of having both low cost and low resistivity.^[2]^ However, Cu foil CC has a high density, and that result in an increase of weight percentage, thus leading to a decrease of energy density. In order to obtain lightweight and flexible electrode, sandwich structure metal/polymer/metal composite CC was fabricated due to the advantages of flexibility and lightweight of polymer. Yun et al. prepared copper coated polymer film CC through magnetron sputtering.^[3]^ Pan et al. fabricated copper coated polyimide film CC through electroless plating method.^[4]^

3D CC has many advantages in improving performance of LIB, including inhibition of Li dendrite formation, alleviation of volume change, and contact well with electrode materials. Yun et al. prepared 3D porous CC via chemical dealloying method, and the 3D porous CC can alleviate the volume change of Li during Li plating/stripping and inhibit the formation of Li dendrites.^[5]^ Chen et al. prepared nano‐holes on the surface of copper CC via ultrasonic chemical etching method, and improved charge/discharge performance of LIB.^[6]^ Silver nanowires was used as CC and achieved a good performance in aqueous LIB.^[7]^ Long et al. prepared 3D copper foil‐powder sintering CC, assembled it with silicon based anode, and achieved a better performance compared with a planar CC.^[8]^

As an engineering plastic, polyimide has been used in various fields, such as microelectronics, high temperature filtration media and aviation. Polyimide (PI) has been applied in LIB in recent years owing to its excellent thermal stability, superior mechanical property, and good electrochemical stability.[^9^, ^10^, ^11^] However, many aromatic PIs are insoluble and therefore it is difficult for manufacturing processes.^[12]^ Electrospinning method is a facile method to obtain nanofiber membranes.^[13]^ Moreover, nanofiber membranes have a lot of advantages, including large specific surface area, high porosity and controllable fiber diameter. PI nanofiber membranes can be prepared by using poly(amic acid) (PAA) as precursor solution to obtain PAA nanofiber membranes, and then go through thermal imidization treatment. This fabrication process uses PAA precursor solution as spinning solution can avoid the difficult of PI manufacturing process. Then after thermal imidization treatment, PI nanofiber membranes can be obtained. Therefore, for many aromatic PIs, electrospinning is a good method to prepare PI nanofiber membranes owing to it can avoid the difficult of shaping PI directly.

In this work, lightweight 3D‐net copper‐plated polyimide CC was prepared through electroless plating method. The structure of Cu‐PI CCs was composed with a PI nanofiber core and copper shell. Three different Cu loading copper‐coated PI CC samples was prepared. Compared with commercial planar Cu foil CC, Cu‐PI CC has lower areal density. Owing to 3D‐net structure, Cu‐PI CC 2 provides high specific surface area and intrinsic porous structure, electrode materials contact better with CC and electrolyte. Therefore, it facilitates electron conduction and Li^+^ transfer and thus improves cycling and rate performance.

## Experimental Details

### Materials

Pyromellitic dianhydride (PMDA) and sodium hydroxide were purchased from Shanghai Aladdin Biochemical Technology Co., Ltd. 4,4’‐diaminodiphenyl ether (ODA), N,N‐dimethylformamide (DMF), copper chloride (CuCl_2_), boric acid, borane dimethylamine complex (DMAB) and 1‐methyl‐2‐pyrrolidinone (NMP) were obtained from Shanghai Macklin Biochemical Co., Ltd. Ethylenediaminetetraacetic acid (EDTA) was purchased from Biosharp. PVDF and a liquid electrolyte of 1 M LiPF_6_ in ethylene carbonate (EC)/diethyl carbonate (DEC) (1 : 1) were obtained from DoDoChem. Li_4_Ti_5_O_12_ (LTO) was obtained from HF‐Kejing. Supper P was supplied by KJ Group.

### Preparation of PI Porous Membranes

PI nanofiber membranes were prepared through electrospinning and thermal imidization. Firstly, the PAA precursor was prepared by dissolving diamine ODA in DMF, then dianhydride PMDA was added into the solution with a molar ratio of 1 : 1 to react with diamine at 30 °C for 6 h. The PAA precursor comprising 25 wt% solids content was prepared for the following electrospinning process.

The PAA membranes were prepared through electrospinning at a distance of 17 cm between the needle tip and collector. The applied voltage was maintained at 22 kV. The speed of the PAA precursor supply was fixed at 0.0005 cm/min. And the rotating speed of the collector was maintained at 220 rpm/min. The prepared PAA membranes were heated following a heating program to obtain PI membranes: 100 °C for 2 h, 200 °C for 2 h, 300 °C for 2 h.

### Preparation of Copper‐Coated PI CC

Copper‐coated PI CC was prepared by electroless copper‐plating method. In order to obtain different Cu loading of PI, different concentration of electroless plating bath was used to conduct electroless plating procedure. In this work, three different Cu loading copper‐coated PI CC samples was prepared. The copper‐coated PI CC was labelled as Cu‐PI CC 1, Cu‐PI CC 2 and Cu‐PI CC 3 with the Cu loading increasing.

The detailed description of electroless plating procedure take Cu‐PI CC 2 as an example. Electroless plating bath was prepared by dissolving 0.125 M EDTA, 0.125 M copper chloride and 0.25 M boric acid in water and then adding sodium hydroxide to adjust to pH 7.0, and 0.25 M DMAB was added into bath solution before use. PI nano‐fiber membrane was soaked in the electroless plating bath until the blue solution turn to colorless. Then copper‐coated PI CC was washed with ultrapure water five times. The copper‐coated PI CC was labelled as Cu‐PI CC 2.

For Cu‐PI CC 1, electroless plating bath was prepared by 0.0625 M EDTA, 0.0625 M copper chloride and 0.125 M boric acid in water and then adding sodium hydroxide to adjust to pH 7.0, and 0.125 M DMAB was added into bath solution before use. For Cu‐PI CC 3, electroless plating bath was prepared by 0.25 M EDTA, 0.25 M copper chloride and 0.5 M boric acid in water and then adding sodium hydroxide to adjust to pH 7.0, and 0.5 M DMAB was added into bath solution before use.

### Cell Assembly

A slurry was prepared of Li_4_Ti_5_O_12_, Super P and PVDF in a mass ratio of 8 : 1 : 1 dissolving in NMP solvent. Then the slurry was pasted on the Cu‐PI CC or the commercial planar Cu foil and dried at 60 °C under vacuum. The active material load of LTO electrode was ca. 1.91 mg cm^−2^. Half cells were assembled using a Li_4_Ti_5_O_12_ work electrode, Celgard 2500 separator and Li metal counter electrode. All the cells were carried out in an Ar‐filled glove box.

### Characterizations

The chemical structures of PI nanofiber membranes were characterized using Fourier transform infrared spectroscopy (FTIR, Thermo Scientific Nicolet 6700). The morphology of the PI nanofiber membranes and the Cu‐PI CCs were observed using scanning electron microscopy (SEM, Hitachi SU8010). The crystal structure of PI membrane and Cu‐PI CCs were characterized by X‐ray diffractometer (XRD, D/MAX‐Ultima IV X‐Ray Diffractometer). The cells were disassembled, and the LTO based electrodes were washed with dimethyl carbonate (DMC) three times and then dried for characterizing their morphologies by SEM. The electrical conductivity of the commercial planar Cu foil CC and Cu‐PI CCs was measured using a four‐point probe tester (ST2258C). The electrochemical impedance spectroscopy (EIS) test was conducted using an electrochemical workstation (CHI760E, Shanghai Chenhua Instrument Co., Ltd) at an amplitude of 5 mV over a frequency range of 10^−2^–10^5^ H_z_.

The cycle performance was measured by discharging and charging at different C‐rates for 200 cycles in a voltage range of 1.0 to 2.5 V. The rate performance was carried out by discharging and charging continuously for five cycles at varying rates, ranging from 0.1 to 5 C and then returning to 0.1 C.

## Results and Discussion

**Figure 1:**
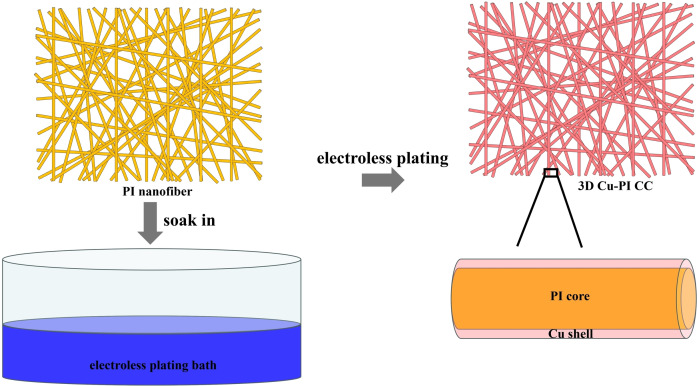
Schematic of the procedure of preparing 3D‐net Cu‐PI CC.

The PI nanofiber membranes were prepared through electrospinning. As depicted in Figure 1, 3D‐net Cu‐PI CC was fabricated by electroless plating method. The reaction of electroless plating process is shown in Equation 1, ^[14]^

(1)

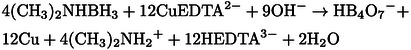




Cu^2+^ was reduced to Cu by DMAB to form Cu layer on the surface of the PI nanofibers. In the electroless plating process, EDTA was used as a coupling reagent, and boric acid was used as buffering agent. After electroless plating, the color of PI nanofiber membranes changed from yellow‐brown to reddish‐brown. It indicates that Cu layer is formed on PI nanofiber membranes.

Areal density of commercial planar Cu foil CC and Cu‐PI CCs were listed in Table 1. The areal density of Cu‐PI CC was increased with the increasing concentration of electroless plating bath. It was indicated that different Cu loading copper‐coated PI CC was prepared successfully by controlling the concentration of electroless plating bath. The areal density of Cu‐PI CC 1 (2.433 mg cm^−2^), Cu‐PI CC 2 (4.804 mg cm^−2^) and Cu‐PI CC 3 (6.635 mg cm^−2^) was lower than commercial planar Cu foil CC (8.846 mg cm^−2^). PI, as one kind of the polymer materials, has a low density. And the porous structure of 3D‐net PI nanofiber membranes can also decrease areal density compared with planar structure materials. Therefore, the lightweight property of Cu‐PI CC should be attributed to both the low areal density of PI core and the 3D‐net structure. As listed in Table 1, the thickness was ca. 11 μm for Cu‐PI CC 1, ca. 14 μm for Cu‐PI CC 2, ca. 21 μm for Cu‐PI CC 3, and 10 μm for commercial planar Cu CC. It can be seen in Table 1, the electrical conductivity was 1.79×10^3^ S/cm for Cu‐PI CC 1, 3.33×10^3^ S/cm for Cu‐PI CC 2, 4.15×10^3^ S/cm for Cu‐PI CC 3, and 5.68×10^5^ S/cm for commercial planar Cu CC. Although the electrical conductivity of Cu‐PI CC was increased with the increasing Cu loading of PI. The electrical conductivity of Cu‐PI CCs was still lower than commercial planar Cu foil CC owing to its 3D structure, and this consistent with Wang et al. reported^[14]^.

**Table 1:** Areal density, thickness and conductivity of CCs.

Sample	Areal density (mg cm^−2^)	Thickness (μm)	Electrical conductivity (S/cm)
Cu foil CC	8.846	10	5.68×10^5^
Cu‐PI CC 1	2.433	11	1.79×10^3^
Cu‐PI CC 2	4.804	14	3.33×10^3^
Cu‐PI CC 3	6.635	21	4.15×10^3^

**Figure 2:**
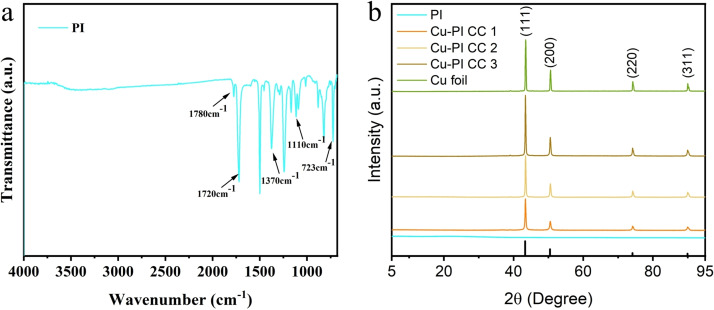
(a) FTIR spectra of PI nanofiber membrane; (b) XRD patterns of PI, Cu‐PI CCs and commercial planar Cu foil.

The chemical structure of PI nanofiber membrane was characterized using FTIR. As shown in Figure 2a, the absorption peaks at 1780, 1720, and 723 cm^−1^ are attributed to asymmetric stretching vibration, symmetric stretching vibration, and bending vibration of the carbonyl group, respectively. The absorption peaks at 1370 and 1110 cm^−1^ are attributed to the stretching vibration of −C−N. It demonstrates that PI nanofiber membranes are prepared successfully after thermal imidization.

The XRD patterns of PI and Cu‐PI CCs was shown in Figure 2b. A broad diffraction peak appears at 22° in PI is attributed to the amorphous peak of PI.^[15]^ Sharp diffraction peaks appear at 43.4, 50.5, 74.2 and 90° in Cu‐PI CCs corresponding to (111), (200), (220) and (311) crystal planes of Cu (PDF#04‐0836). It indicates that Cu layer was formed successfully on surface of PI nanofiber membrane after electroless plating.

**Figure 3:**
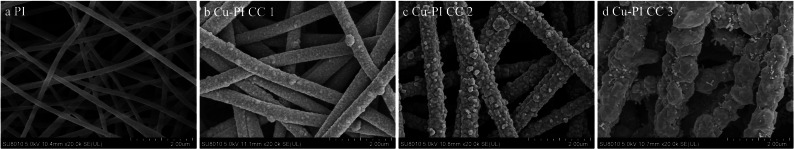
SEM images of (a) PI nanofiber membrane; (b) Cu‐PI CC 1; (c) Cu‐PI CC 2; (d) Cu‐PI CC 3.

In order to explore the structure of Cu‐PI CCs, the morphology of Cu‐PI CCs was observed by SEM. As Figure 3a shows, PI nanofiber membrane was successfully prepared through electrospinning. After electroless plating, the surface of PI nanofiber was coated a layer of Cu particles, as shown in Figures 3b–d. The size of Cu particles increased with the increasing concentration of electroless plating bath. And Figures 3b–d also showed that Cu‐PI CCs were successfully obtained with different Cu loading by controlling concentration of electroless plating bath. The Cu particles were formed on the surface of PI nanofibers, not just on the surface of membrane. These nanofibers with PI core and Cu shell are heaped together to form a 3D‐net structure Cu‐PI CCs. Cu shell as electrical conductivity layer to provide paths for electrons conduction.

**Figure 4:**
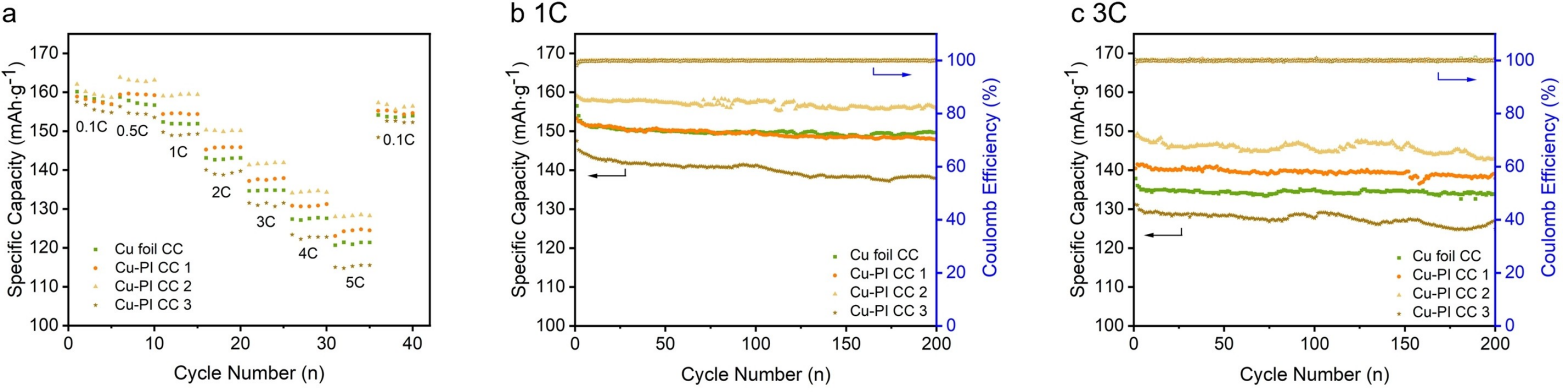
(a) Rate capability; (b) Cycle performance at 1 C; (c) Cycle performance at 3 C of LTO electrode assembled with Cu‐PI CCs and Cu foil CC.

**Figure 5:**
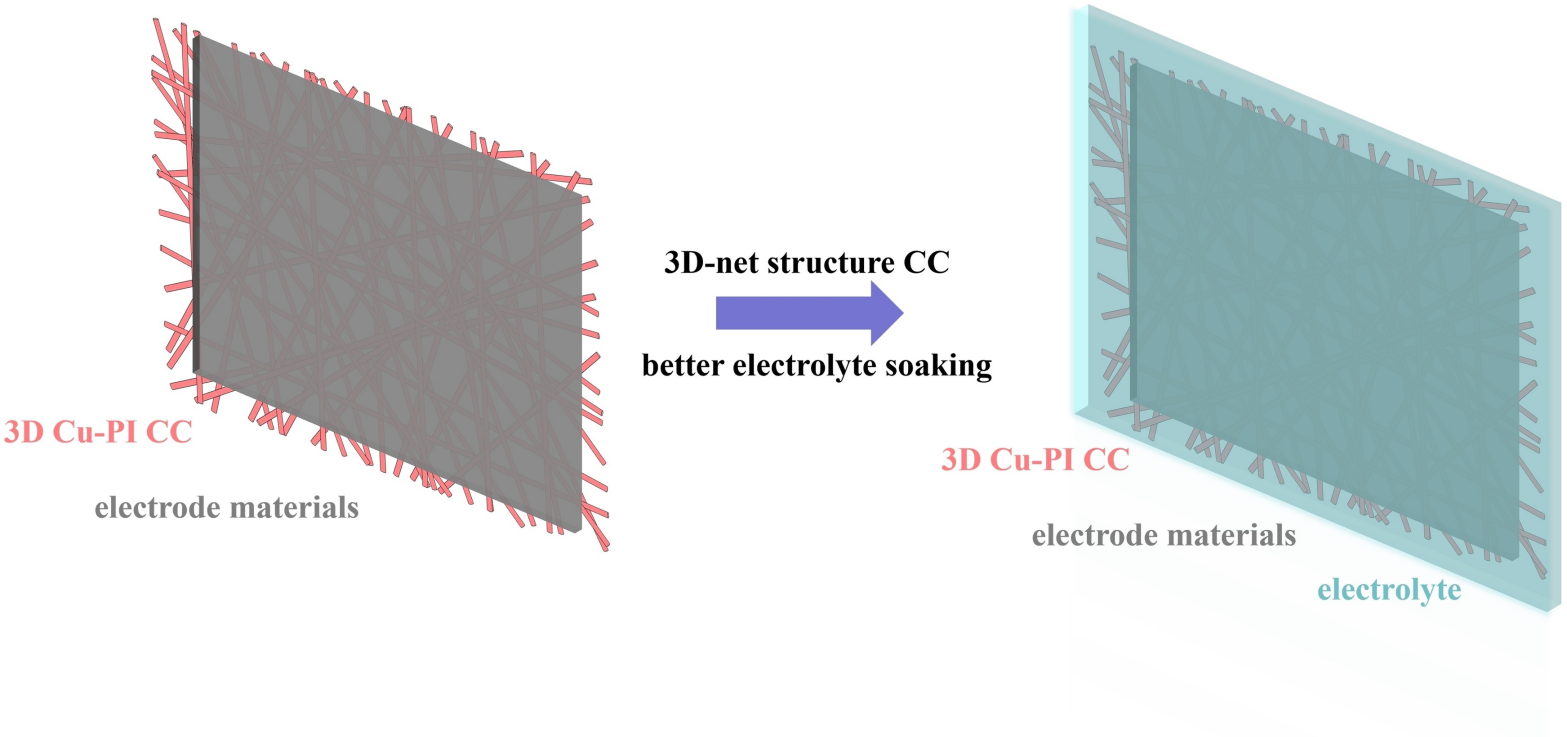
Schematic of 3D‐net structure Cu‐PI CC 2 assembled with electrode materials.

As shown in Figure 4a, the cells assembled with the Cu‐PI CC 2 exhibited excellent rate capacity than those assembled with the Cu foil CC. Owing to the sufficient permeation of electrolyte among electrode materials and that can facilitate Li^+^ transfer between active materials and electrolyte, as Figure 5 shown. Therefore, Cu‐PI CC 2 exhibited superior performance, especially in the high‐rate range. The cells assembled with the Cu‐PI CC 3 exhibited a poor performance than Cu foil CC. It should be attributed to its thicker thickness, and that could extend the ion transport distance.^[16]^

Figures 4b–c shows the cycle performance of the cells assembled with the Cu‐PI CCs and commercial planar Cu foil CC at different rates. All the cells were tested at 1 C. After 200 cycles at 1 C, the cells assembled with the Cu‐PI CC 2 exhibited superior performance than those assembled with the commercial planar Cu foil CC, Cu‐PI CC 1 and Cu‐PI CC 3. The cells achieved a discharge specific capacity of 156 mAh g^−1^ for Cu‐PI CC 2, 148 mAh g^−1^for Cu‐PI CC 1, 140 mAh g^−1^ for Cu‐PI CC 3 and 149 mAh g^−1^ for Cu foil CC after 200 cycles at 1 C. The cycle performance of Cu‐PI CC 2 was superior than that of commercial planar Cu foil CC owing to the sufficient permeation of electrolyte, and that can facilitate Li^+^ transfer between active materials and electrolyte, as Figure 5 shown. The cells achieved a discharge specific capacity of 145 mAh g^−1^ for Cu‐PI CC 2, 139 mAh g^−1^for Cu‐PI CC 1, 129 mAh g^−1^ for Cu‐PI CC 3 and 135 mAh g^−1^ for Cu foil after 200 cycles at 3 C.

Compared with commercial planar Cu foil CC, Cu‐PI CC 2 exhibit a better performance, and Cu‐PI CC 1 has a similar performance. However, Cu‐PI CC 3 show a poor performance than commercial planar Cu foil CC, it should be attributed to its thicker thickness, and that could extend the ion transport distance,^[16]^ and thus lead to a lower specific capacity.

With increasing Cu loading, the performance of Cu‐PI CC improved first, then become worse than commercial planar Cu foil CC. It should be attributed to two reasons. On the one hand, the electrical conductivity was improved with increasing Cu loading, and lead to a better performance in electrons transfer. On the other hand, the thickness of Cu‐PI CC was increased with increasing Cu loading, as a result, the ion transport distance extended, especially for Cu‐PI CC 3, and it lead to an adverse effect on battery performance.

**Figure 6:**
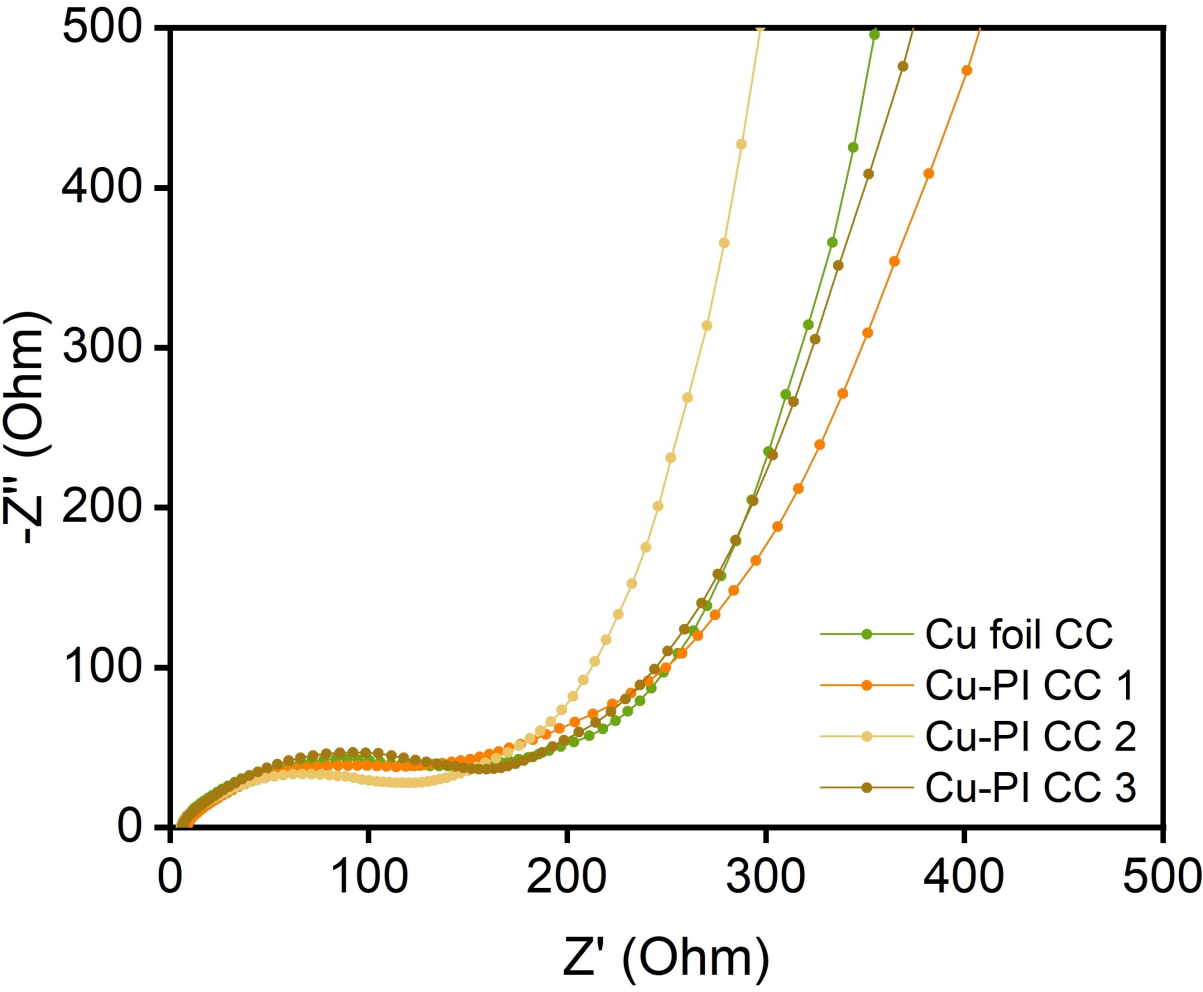
Nyquist plots of cells assembled with Cu foil CC and Cu‐PI CCs.

Figure 6 shows the Nyquist plots of the cells assembled with Cu foil CC and Cu‐PI CCs. The Nyquist plots of all these cells include a semi‐circle and a linear portion. The intercept on the real axis was attributed to the ohmic resistance (R_e_) of the ionic resistance of electrolyte, the ohmic resistance of the electrode layer and the CC, and the contact resistance between electrode layer and CC. The width of the semi‐circle was attributed to the charge transfer resistance (R_ct_) (Table 2).

**Table 2:** EIS fitting results of cells assembled with Cu foil CC and Cu‐PI CCs.

	R_e_ (Ω)	Rct (Ω)
Cu foil CC	4.62	133.3
Cu‐PI CC 1	7.40	122.3
Cu‐PI CC 2	4.72	108.1
Cu‐PI CC 3	5.38	134.2

The R_e_ of Cu‐PI CC 2 (4.72 Ω) was similar to Cu foil CC (4.62 Ω). However, Cu‐PI CC 1 (7.40 Ω) was higher than Cu foil CC. It may due to the Cu‐PI CC 1 have a very lower electrical conductivity than other Cu‐PI CCs. Although all the Cu‐PI CCs have a lower electrical conductivity than Cu foil CC, as shown in Table 1. Compared with Cu foil CC, Cu‐PI CC 2 show a similar value of R_e_ owing to its 3D‐net structure lead to a large interfacial contact area.^[4]^

The R_ct_ of Cu‐PI CC 2 (108.1 Ω), Cu‐PI CC 1 (122.3 Ω) is lower than Cu foil CC (133.3 Ω). The lower R_ct_ value lead to an increasing specific capacity of LIB.^[17]^ Its 3D‐net structure provide superior electrolyte permeation and sufficient connection area for electrode layer and CC, as a result, its electrochemical performance is better than Cu foil CC. The R_ct_ of Cu‐PI CC 3 (134.2 Ω) is higher than Cu foil CC, therefore, its specific capacity is lower than Cu foil CC. And its thicker thickness also extend the ion transport distance, thus has an adverse effect on its specific capacity.

Providing sufficient paths for Li^+^ and electrons can improve the performance of LIB.^[2]^ Compared with commercial planar Cu foil CC, Cu‐PI CC 2 can provide larger and more sufficient specific surface area due to its 3D‐net structure. Therefore, on the one hand, owing to the larger specific surface area of 3D ‐net structure, Cu‐PI CC 2 contact better with electrode material layer and that can facilitate electrons conduction. On the other hand, the electrolyte can permeate through the electrode material layer and the 3D‐net Cu‐PI CC 2, as a result, Cu‐PI CC 2 provide larger contact area for Li^+^ transfer between electrolyte and electrode material. Therefore, the performance of the cells assembled with Cu‐PI CC 2 improved compared with commercial planar Cu foil CC. This design strategy of 3D‐net Cu‐PI CC may provide a method to prepare novel lightweight LIB with enhanced performance.

## Conclusions

In summary, 3D‐net Cu‐PI CC was successfully prepared through electroless plating method. Three different Cu loading copper‐coated PI CC samples was prepared and compared. The lightweight Cu‐PI CC 2 possess a low areal density of 4.804 mg cm^−2^ and a 3D‐net structure. 3D‐net structure of Cu‐PI CC 2 provides large specific surface area to contact better with electrode materials and that facilitate electrons conduction. Moreover, Cu‐PI CC 2 provides sufficient contact area for Li^+^ transfer between electrolyte and electrode materials owing to its 3D‐net structure can facilitate electrolyte permeation. The half cells assembled with Cu‐PI CC 2 exhibited excellent cycling and rate performance.

## Conflict of Interests

There are no conflicts to declare.

1

## Supporting information

As a service to our authors and readers, this journal provides supporting information supplied by the authors. Such materials are peer reviewed and may be re‐organized for online delivery, but are not copy‐edited or typeset. Technical support issues arising from supporting information (other than missing files) should be addressed to the authors.

Supporting Information

## Data Availability

The data that support the findings of this study are available in the supplementary material of this article.
