# Supplementary material for: Lightweight 3D‐net Copper‐Plated Polyimide Current Collector for Lithium‐Ion Batteries
Source: ChemistryOpen. 2024 Nov 26;14(4):e202400018. doi: 10.1002/open.202400018 (PMC11973500; doi:10.1002/open.202400018)
Supplement: Supplementary file 1 — Supporting Information [file OPEN-14-e202400018-s001.pdf]

Chemis-
tryOpen

Supporting Information

Lightweight 3D-net Copper-Plated Polyimide Current Collector for Lithium-Ion Batteries

Tingyu Song and Yonggang Min*

Supporting Information

Lightweight 3D-net copper-plated polyimide current collector for lithium-ion batteries

*Tingyu Song and Yonggang Min**

T. Song and Y. Min

School of Materials and Energy, Guangdong University of Technology, Guangzhou 510006, China

E-mail: ygmin@gdut.edu.cn

Table S1. Concentration of electroless plating bath

	EDTA	copper chloride	boric acid	sodium hydroxide	DMAB
Cu-PI CC 1	0.0625M	0.0625M	0.125 M	adjust pH	0.125 M
Cu-PI CC 2	0.125 M	0.125 M	0.25 M	adjust pH	0.25 M
Cu-PI CC 3	0.25 M	0.25 M	0.5 M	adjust pH	0.5 M

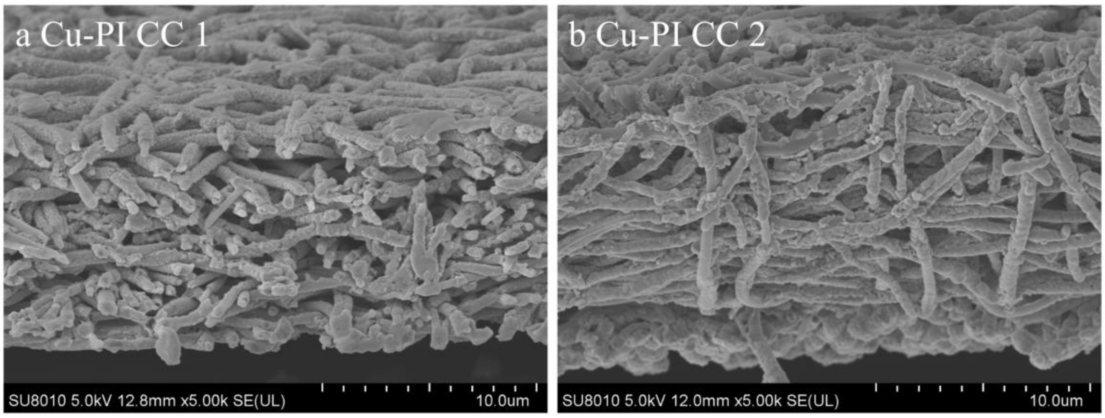

Figure S1. Cross-sectional SEM images of (a) Cu-PI CC 1; (b) Cu-PI CC 2.

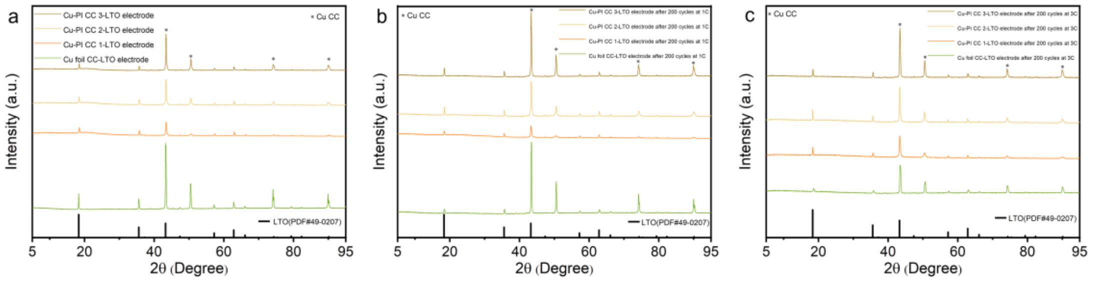

Figure S2. XRD patterns of Cu CC-LTO and Cu-PI CC-LTO electrodes (a) before cycles; (b) after 200 cycles at 1C; (c) after 200 cycles at 2C; (d) after 200 cycles at 3C.

The structure of the electrode was characterized by XRD before and after cycling test. The LTO electrodes

were prepared by using commercial planar Cu foil CC, Cu-PI CC 1, Cu-PI CC 2 and Cu-PI CC 3. As Figure S2a shows, in all electrodes, the peaks at 18.3, 30.2, 35.6, 37.2, 43.2, 47.4, 57.2, 62.8, 66.1, 74.3, 75.4, 79.3, 82.3, 90.1° can be ascribed to the (111), (220), (311), (222), (400), (331), (333), (440), (531), (533), (622), (444), (551), (731) planes of active material spinel LTO (PDF#49-0207). The peaks at 43.4, 50.5, 74.2 and 90° in all electrodes corresponding to (111), (200), (220) and (311) crystal planes of Cu current collector, because X-rays can penetrate the electrode materials layer. Figure S2b-c shows the XRD patterns of LTO electrode after cycling tests, no obvious different change between the LTO electrodes assembled with different CCs after cycling tests.

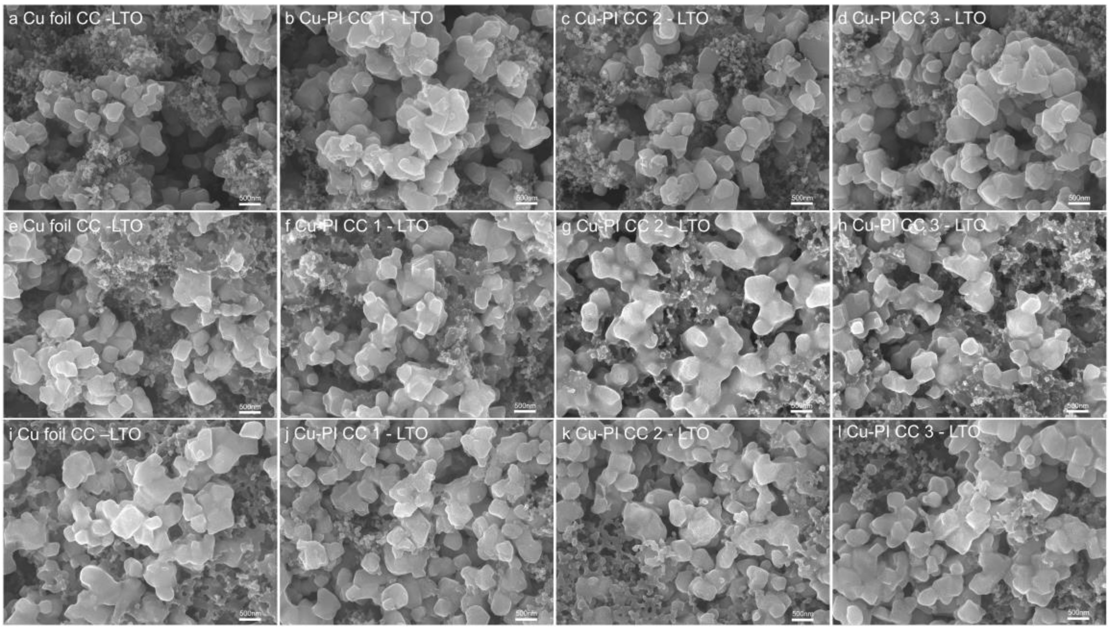

Figure S3. SEM images of Cu foil CC-LTO and Cu-PI CC-LTO electrodes (a-d) before cycles; (e-h) after 200 cycles at 1C; (i-l) after 200 cycles at 3C.

The morphology of LTO electrode with Cu foil CC and Cu-PI CCs were tested by SEM before and after cycling tests. Figure S3a-d shows the Cu foil CC-LTO and Cu-PI CC-LTO electrodes before cycling test. Figure Se-l show the Cu foil CC-LTO and Cu-PI CC-LTO electrodes after cycling tests. Compared with the electrodes before cycling test, all the electrodes were covered with a layer of SEI film.
